# Cortical representation of different taste modalities on the gustatory cortex: A pilot study

**DOI:** 10.1371/journal.pone.0190164

**Published:** 2017-12-27

**Authors:** Anna Prinster, Elena Cantone, Viviana Verlezza, Mario Magliulo, Giovanni Sarnelli, Maurizio Iengo, Rosario Cuomo, Francesco Di Salle, Fabrizio Esposito

**Affiliations:** 1 Biostructure and Bioimaging Institute, National Research Council, Naples, Italy; 2 Section of ENT, Department of Neuroscience, "Federico II" University, Naples, Italy; 3 Gastroenterology Unit, Department of Clinical and Experimental Medicine, “Federico II” University, Naples, Italy; 4 Department of Medicine, Surgery and Dentistry, University of Salerno, Baronissi (Salerno), Italy; Duke University School of Medicine, UNITED STATES

## Abstract

**Background:**

Right insular cortex is involved in taste discrimination, but its functional organization is still poorly known. In general, sensory cortices represent the spatial prevalence of relevant features for each sensory modality (visual, auditory, somatosensory) in an ordered way across the cortical space. Following this analogy, we hypothesized that primary taste cortex is organized in similar ordered way in response to six tastes with known receptorial mechanisms (sweet, bitter, sour, salt, umami, CO_2_)_._

**Design:**

Ten normal subjects were enrolled in a pilot study. We used functional magnetic resonance imaging (fMRI), a high resolution cortical registration method, and specialized procedures of feature prevalence localization, to map fMRI responses within the right insular cortex, to water solutions of quinine hydrochloride (bitter), Acesulfamate K (sweet), sodium chloride (salt), mono potassium glutamate + inosine 5' mono phosphate (Umami), citric acid (sour) and carbonated water (CO_2_). During an fMRI scan delivery of the solutions was applied in pseudo-random order interleaved with cleaning water.

**Results:**

Two subjects were discarded due to excessive head movements. In the remaining subjects, statistically significant activations were detected in the fMRI responses to all tastes in the right insular cortex (p<0.05, family-wise corrected for multiple comparisons). Cortical representation of taste prevalence highlighted two spatially segregated clusters, processing two and three tastes coupled together (sweet-bitter and salt-umami-sour), with CO_2_ in between.

**Conclusions:**

Cortical representation of taste prevalence within the right primary taste cortex appears to follow the ecological purpose of enhancing the discrimination between safe nutrients and harmful substances.

## Introduction

The mechanisms underlying perception, from peripheral transduction to central neural processing of sensory information, have been studied in detail in the visual, auditory and somatosensory systems, where key physical properties of the external stimuli are analyzed by specific receptors in the peripheral sense organs and maintained in the sensory cortex. To facilitate discrimination, neuronal columns responsive to similar stimulus features are clustered, and response properties vary smoothly, across the cortical sheet [1–4].

While quantitative dimensions like position or frequency are naturally well suited to be represented in spatially ordered cortical locations, stimulus features relevant for the chemical senses (olfaction and taste) do not include continuous physical properties other than magnitude (or intensity). Instead, the quality of a chemical stimulus is categorized according to a chemical composition that does not exhibit variations along a physical dimension common to different substances.

The olfactory system employs spatial segregation of sensory inputs to encode the quality of odorants [5, 6], but the anatomical projections from the olfactory bulb to the cortex do not necessarily map adjacent peripheral stations to contiguous cortical locations [7]. Similarly to olfaction, the quality of taste stimuli does not implicitly provide an anatomical criterion to expect that a given tastant is preferentially represented in any given region of the cortex. Nonetheless, a spatially segregated and ordered cortical representation of odorants' or tastants' qualities, as opposed to a randomly distributed representation, may still result from (genetically determined) stereotyped neural circuits in specific regions, thus explaining innate ecological behaviors [7]. Particularly, cortical taste processing may tune each taste domain to identify specific nutrients, harmful substances, or chemicals associated with physiological functions [8], thereby, an ordered cortical organization for taste would facilitate the discrimination among tastants with mechanisms similar to those observed in other sensory cortices. Humans perceive five distinct basic tastes: sweet, umami, bitter, sour, and salt. Recently, a dedicated receptorial mechanism for CO_2_ has been discovered, sub serving the taste of carbonation [9]. Taste perception begins in the tongue, where three types of gustatory papillae (circumvallate, foliate, and fungiform papillae) are found [10], follows multiple ascending pathways in the brainstem [11], and activates the Primary Taste Cortex (PTC). The PTC is located in the insular cortex near the somatotopic region for the tongue; secondary taste areas are located in the opercula. In particular, the right insular cortex and the right frontal operculum are primarily involved in the discrimination of tastes [12, 13].

Previous studies substantially failed to demonstrate a chemotopic organization of taste cortical processing in humans, presumably due to the inter-individual anatomical variability of the PTC responses [14], or because stimulus properties other than taste quality were taken into account, such as physiological significance, pleasantness or taste intensity [15]. Moreover, some animal imaging reports argue for chemotopy in mammalian brain cortex [16–18], but a more recent imaging report argues against this and shows no spatial organization of taste sensitivity in mouse cortical regions [19]. In this pilot study, we stimulated all six tastes with known receptorial mechanism in ten healthy volunteers, and investigated the quality coding of the different tastes.

We used fMRI with a high resolution cortical registration method to improve the spatial correspondence across individual brains [20, 21], and specialized analytical tools for cortical prevalence mapping, to identify best stimulus response patches in the PTC that could be assigned to different tastes. The use of best-stimulus maps has been previously adopted to demonstrate tonotopy in the human primary auditory cortex [22]. Accordingly, we hypothesized the existence of a common chemotopic pattern suggesting a functionally-oriented organization of taste processing, fundamental for the discrimination between safe nutrients and possibly harmful substances.

## Materials and methods

### Subjects and behavioral test

Ten adult healthy volunteers (5 males and 5 females, mean age ± standard deviation 24,45 ± 2,1) not reporting any olfactory, gustatory, neurological or psychiatric disorder, and free from the use of any medication or smoking, took part in the study. The study was approved by the Ethics Committee of the “Federico II” University of Naples (Protocol Number 231/07) and written consent was obtained by all participants.

Solutions of 10 mM of quinine hydrochloride (bitter), 30mM of Acesulfamate K (sweet), 120mM sodium chloride (salt), 30mM of mono potassium glutamate +0,5mM inosine 5' mono phosphate (Umami), 50mM citric acid (sour) and carbonated water (containing 1585 ppm of CO_2_) were used.

Prior to fMRI examination, all subjects underwent behavioral tests to verify that all the subjects were able to discriminate among the different tastes and to correctly identify all the solutions that were then administered during fMRI experiment. The tests were based on the Visual Analogue Scale (VAS) questionnaire, which includes questions on identification and intensity of each solution (0 = Not perceived, 100 mm = maximum perceived). Subjects were also instructed not to eat or drink 1 h before testing, in order to avoid chemosensory desensitization. Exclusion criteria for the study were: swallowing difficulties or other neurologic deficits, structural or functional oral peripheral abnormalities, and food allergies or intolerance to any of the tastants used in the samples.

Behavioral tests took place in a silent room at warm temperature (20°C). All solutions used in the study were colorless and retained in similar plastic 5 ml tubes with a protective cap to prevent the loss of CO_2_ and numbered with a key that was decoded only at the end of each session. The solutions were stored in refrigerator at cool temperature of 4°C and presented to participants less than 5 minutes before the test. A maximum of two subjects performed the evaluation at the same time in order to keep a silent atmosphere. We adopted a questionnaire to evaluate the ability to recognize the samples and before starting the analysis the observers checked that the questionnaire was well understood by all subjects after they had read it.

Each solution was administered twice in random order and the still water was used as a control substance. After every stimulus, subjects were invited to rinse their mouth with water and to eat unsalted crackers in order to recover a neutral taste sensation on their tongue [23].

### fMRI experiment

**Fig 1:**
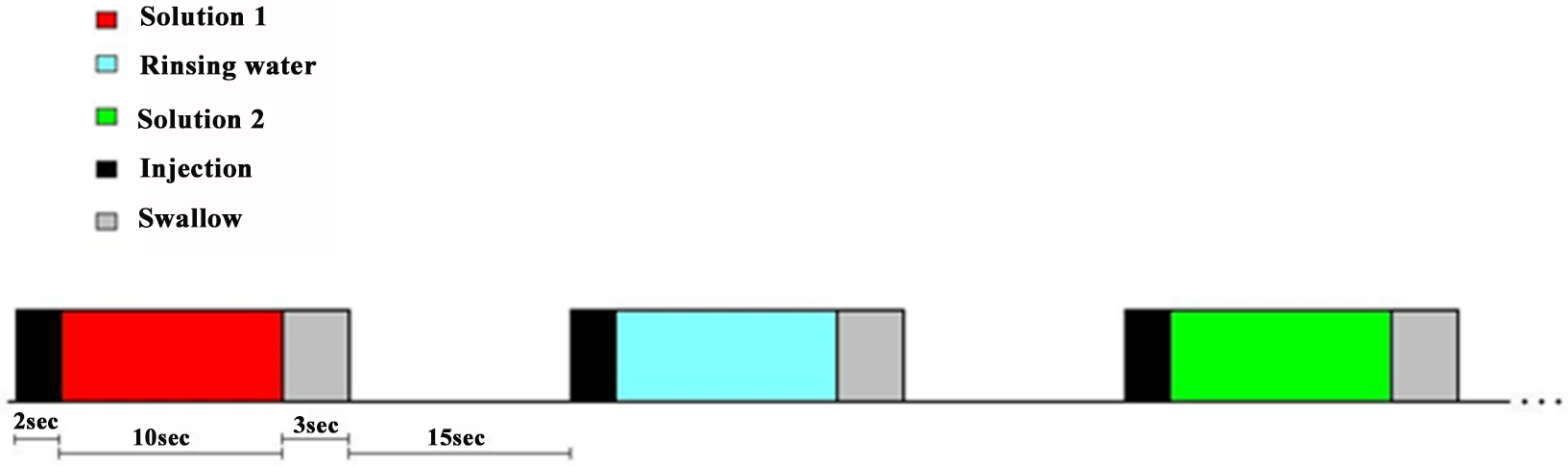
Solutions administration protocol. Taste event consisted of the delivery of 5ml of one solution over 2 sec (2,5 ml/sec injection speed) announced by an auditory cue and followed by a gustatory period of 10 sec. Another auditory cue announced the swallowing of the solution (3 sec) and was followed by a rest period of 15 seconds before the administration of rinse water with the same modality.

The fMRI experiment consisted in the administration and tasting of the six tastants in a pseudo-randomized order while subjects were lying in the magnet. All tastants were stored at controlled low temperature (4°C) prior to examination to keep the same starting condition for all the subjects. Solutions were brought to the site of experiment in a polysterene box and the filling of the syringes and all the experiments were carried out after the tastants reached the room temperature. The six tastants plus rinsing water were administered via four medical injectors (Spectris Solaris, Medrad_TM_) equipped with two syringes each, electronically controlled through a digital I/O device connected to a laptop. Each tastant plus rinsing water was delivered through a dedicated line to avoid cross-contamination of stimuli. The seven lines were pulled together and fixed to the midline of the lips thus avoiding shifting of the tubes. Synchronization with fMRI acquisitions and stimuli delivery were controlled using Presentation® software (www.neurobs.com). Each taste event consisted of the delivery of 5ml of one solution over 2 sec (2,5 ml/sec injection speed) announced by an auditory cue and followed by a gustatory period of 10 sec during which the solution was distributed all over the mouth to avoid the lateralization of the stimulation. Another auditory cue announced the swallowing of the solution (3 sec) and was followed by a rest period of 15 seconds before the administration of rinse water with the same modality (see Fig 1). The alternation of stimuli and water prevented physical and perceptual overlapping among the different tastes. To avoid potential adaptation effects, prior to the execution of the exam, we verified that all the subjects were able to rinse properly after tasting the solution even in the supine position. Each tastant was delivered four times in a pseudo-randomized order according to Table 1.

**Table 1:** Scheme of alternation among tastes.

Seq A	Seq B	Seq C	Seq D
CO_2_	Bitter	Bitter	Sweet
Umami	Sweet	CO_2_	Bitter
Sweet	Salty	Sweet	Sour
Sour	CO_2_	Umami	CO_2_
Bitter	Sour	Sour	Salty
Salty	Umami	Salty	Umami

The six tastants (sweet, bitter, sour, salty, umami and CO_2_) was delivered four times following the sequence A-B-C-D during the fMRI experiments. After each tastant rinse water was delivered to avoid interference among them. Total scan duration was 24 min during which a total of 720 dynamic scans were acquired.

Total fMRI scan duration was 24 min with the acquisition of 720 dynamic scans and total beverage volume of 240ml. Images were acquired on a 1,5T Achieva scanner (Philips Medical System, Eindhoven, Holland) with an EPI single-shot acquisition (TE = 35ms, TR = 2000ms, 64x64 matrix, in-plane voxel 3,59 x 3,59 mm, 22 slices 4 mm thick and 1 mm gap). Patients were firmly fixed with foam pads to minimize head movements during swallowing. At the end of the fMRI acquisition, a T1-weighted 3D TFE SENSE volume was acquired (sagittal; matrix 256 x 256; FOV 220 x 220 mm; 181 slices; slice thickness 1.2 mm; no gap; in-plane voxel size 0,86mm x 0.86mm; flip angle 10°; TR 8 ms; TE 2.2 ms, 2 averages) to provide the anatomical reference for the functional scan and to allow high resolution cortical realignment of subjects' brains.

### Data analysis

Behavioral data were tabulated and the difference in sensory intensity among the different taste stimuli was tested by a repeated measures ANOVA analysis with the Tukey post test. The level of significance was chosen as p < 0.05.

fMRI data were preprocessed using SPM8 (Wellcome Department of Imaging Neuroscience, University College, London, www.fil.ion.ucl.ac.uk/spm) running under Matlab R2011b (Mathworks Inc.). After correcting for slice timing, data were realigned to the first volume and motion corrected. All dynamic scans were normalized to the MNI space and smoothed with a Gaussian filter of 8mm full-width at half-maximum (FWHM). A General Linear Model (GLM) was created with 6 regressors, one for each tastant, a regressor of non-interest for the auditory cues (announcing the start of liquid injection and releasing swallowing) and two regressors of non-interest for swallowing and rinsing water period. In the model, movement regressors were also added, and an high pass filter of 128 sec was applied prior to statistical analysis. The model included the canonical Hemodynamic Response with time derivatives to account for shift in the taste activation responses. For each patients 6 contrast maps were obtained, looking at the statistical effect produced by each tastant (Umami, Sweet, Bitter, Acid, Salty and CO_2_) versus rest. These contrast images were fed in a random effect group analysis to statistically evaluate the main effect of group for each taste. Random effect analysis was performed adding a mask of the insular cortex and thresholded at a value of p<0.05 FWE corrected at cluster level.

Contrast maps for each patients and for each tastant were also obtained with the same procedure leaving EPI volumes in their original space and were imported together with each anatomical volume in BrainVoyager™ QX, (Brain Innovation, Maastricht, The Netherlands) for cortical alignment. We applied a high-resolution cortical alignment method to improve the spatial correspondence across brains and look at the group response in the insular region.

### Cortical alignment and prevalence mapping

For each subject, the cortical surface was reconstructed as a three-dimensional mesh from the segmented Talairach-transformed anatomical images. To represent topological taste activations on the insular cortex, individual GLM beta maps for each contrast were first projected onto the individual cortical mesh and then averaged across subjects subsequent to the cortical-based alignment (CBA) procedure [20]. For each separate hemisphere, CBA was used to map the individual cortical surface vertices to an average cortical surface, to be used as internal common target surface for representing maps. For each single vertex of the common target surface, we averaged the (anatomically aligned) beta weight values resulting from the individual GLM analyses in the voxel space, after projecting to the individual cortical mesh. The use of a CBA approach for group-level analysis was chosen to maximally preserve the anatomical specificity of each individual beta map [21]. We applied CBA as implemented in BrainVoyager QX 2.8.2, using all default parameter settings and choosing the "moving target" approach, according to which, all individual brains are iteratively aligned to a dynamically updated average brain only based on the cortical folding patterns. The cortical folding patterns are quantitatively obtained by the curvature values calculated from the cortex shape (mesh) and therefore represent the alternation of gyri and sulci of the brain. The matching procedure essentially aligns corresponding gyri and sulci across individual’s brains with the result of reducing the anatomical inter-subject variability see also Goebel et al. [21] for more details about the method, and, e.g., Frost et al. [20, 24] and Herdener et al. [25] for recent applications of CBA in fMRI data analyses).

A common mask for the insula was obtained from the anatomical high resolution acquisition of one subject using Brain Parser v5.0 [26] and then projected on the target brain to define the space of prevalence mapping quantification.

In order to quantify topological taste prevalence and the spatial relations among tastes within the insular cortex, for each vertex on the target mesh, we calculated the number of standard deviation of the beta value from the mean beta of the entire insular mask (z-score). This calculation was iterated across all vertices of the insula, for all tastes and for all individual subjects' and group averaged beta maps. Then, we ranked the z-score values from all tastes at each vertex of the mesh and assigned a different color code to the vertex depending on the highest z-score value among all taste stimuli. This way, we intended to emphasize the relative prevalence of the stimulus across the entire insula, regardless of possible overall differences in the amount of activity elicited in the insular cortex. To further illustrate the taste preference at the cluster level, all obtained single-vertex rankings for all tastes were normalized between 0 (worse preference) and 1 (best preference) and averaged across clusters with homogenous taste preference.

To further verify that different individuals exhibited similar responses to each and any specific tastant compared to the entire group of subjects, we used the individual beta maps as responses to each tastant as well as the individual preference maps as responses to any tastants, and calculated the spatial correlation coefficients with the corresponding maps obtained from the group analysis. For individual beta maps, we used Pearson correlation coefficients. For individual preference maps, we used Spearman correlation coefficient because, as anticipated, the information in preference maps is stored in the rank of each tastant.

## Results

**Fig 2:**
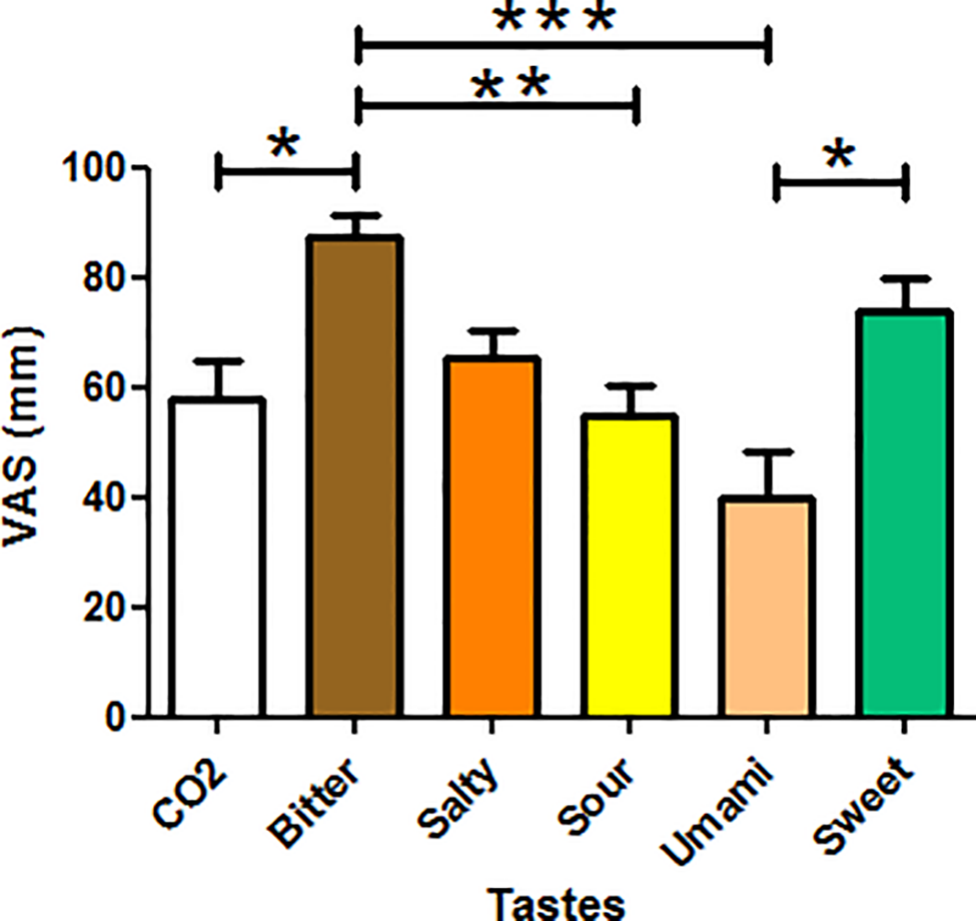
Results of the VAS questionnaire analysis. The average taste perception is reported for all tastes with the significant differences between them at ANOVA (p<0.05).

The average percentage of taste identification was 88% for all tastes (min 75% bitter and max 100% salt); at ANOVA taste perception was reported as stronger for Bitter *vs* Umami (87±13 *vs* 40±28 p-value <0.05); Bitter *vs* Sour (87±13 vs 55±23 p-value <0.05); Bitter vs CO_2_ (87±13 *vs* 58±23 p-value <0.05) and weaker for Umami *vs* Sweet (40±28 ± 74±19 p-value <0.05), see Fig 2 for a complete analysis of VAS results.

Two of the ten subjects were excluded from the image analysis because of excessive and repeated movements in swallowing during fMRI scanning. In this two cases head movements during scan were either more than 1 pixel size in head translation or more than 1degree in head rotation.

In Table 2 the Montreal Neurological Institute (MNI) coordinates of the peak activation of all tastes resulted from the random effects group analysis on the eight subjects are tabulated together with the size and T values of the clusters (p<0,05 FWE corrected at cluster level).

**Table 2:** Main effect of tastes in the right insular cortex.

	MNI Coordinates				
	Right				
	x	Y	z	cluster size	T
Sweet	40	-24	4	86	5,06
Bitter	34	-4	-4	103	4,86
CO_2_	38	-6	0	197	5,4
Salty	36	-8	0	358	5,96
Umami	28	4	14	200	5,28
Sour	32	0	12	261	6,26

Main effect of tastes in the right insular cortex as a result of the within-group random effect analysis (8 subjects). Coordinates of the peak activation cluster in the MNI normalized space, size and T values of the cluster (p<0.05 FWE corrected at cluster level) are reported. Tastes are ordered from posterior to anterior insula.

**Fig 3:**
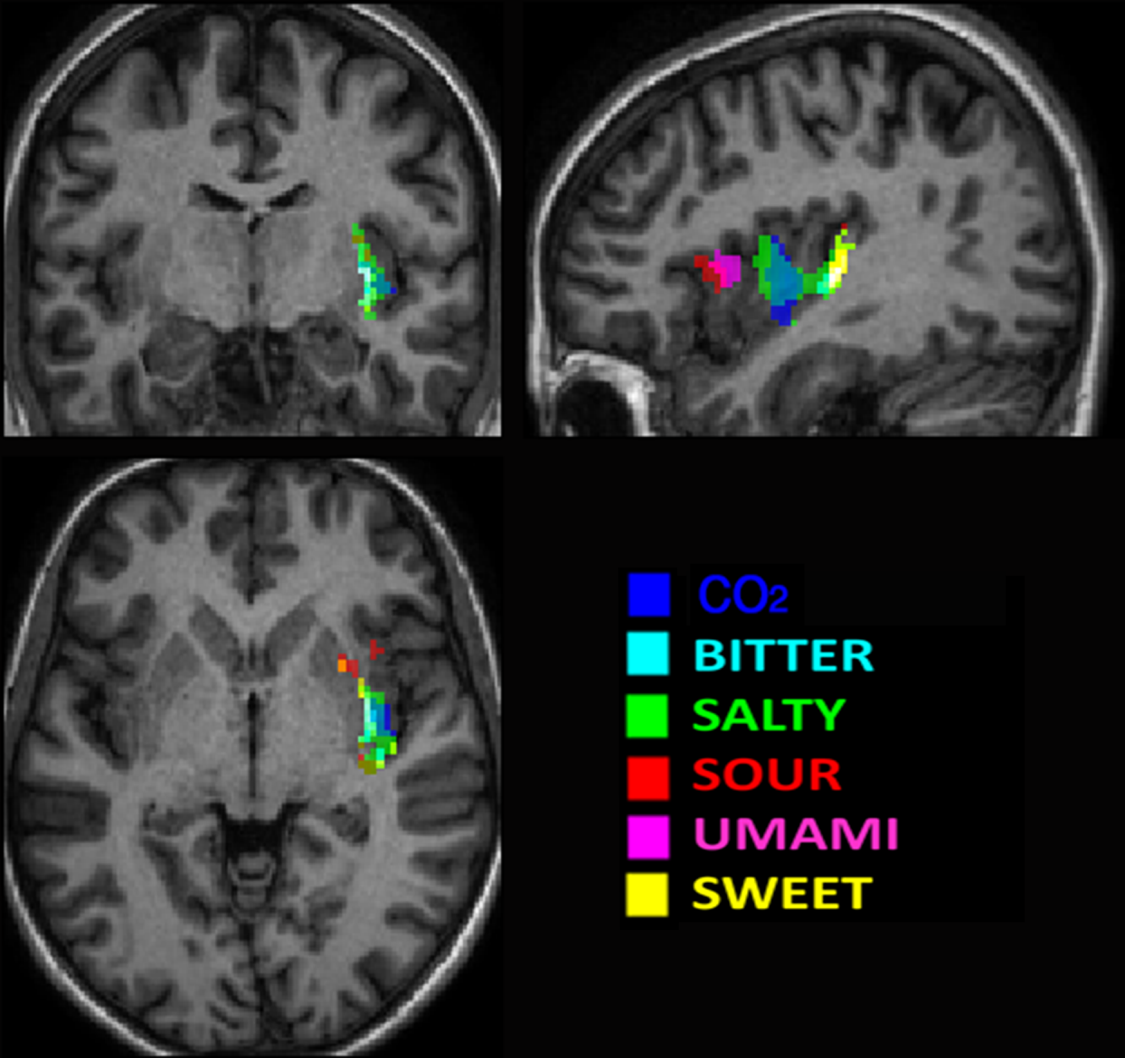
Map of the main effects of tastes in the right insular cortex. Color coded clusters of the within group Random Effect analysis for the different tastants overlapped to a high resolution T1 image after the inclusion of a mask of the right insular cortex (p<0.05 FWE corrected at cluster level). Evidence of a spatial distribution of the six principal tastes is clearly shown even with the overlap of some tastants.

Although our study was designed to stimulate presumably the entire oral cavity to avoid a possible lateralization of the stimulation (the subjects held in their mouth 5 ml of solutions for 10 sec distributing them all over the mouth), we found a statistically significant activation only in the right hemisphere. A selective activation of the right hemisphere is in accordance with the results of Schoenfeld et al. [14], albeit evidence of bilateral insular processing of tastes has been reported in the literature [13, 27–29] and some differences in the role of the two hemispheres have been previously suggested [30]. In Fig 3 maps of the significant clusters of the Random Effect analysis are color coded for the different tastants and are overlapped to a high resolution T1 image after applying a mask on the right insular cortex. Evidence of a spatial distribution of the six principal tastes is clearly shown albeit an overlapping of some tastants is also manifest due to the used method of analysis.

**Fig 4:**
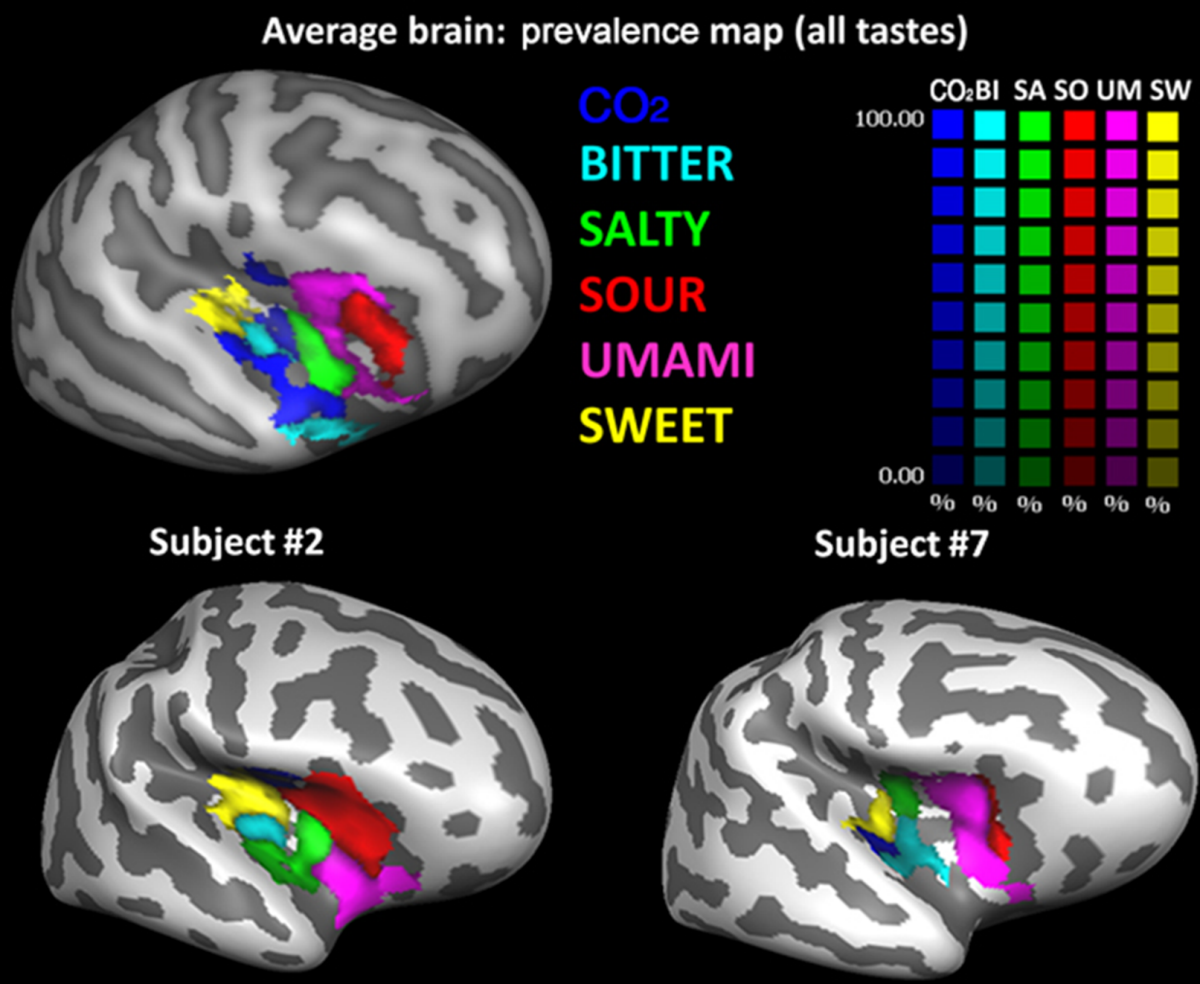
Cortical surface representation of right insular activation of the standardized five basic tastes and CO_2_. In these maps, each vertex of the CBA alignment (top row) is colored with the taste that has the biggest response averaged across all subjects and its color intensity indicate how strong is the taste prevalence (a 100% value indicate that the taste prevalence is twice the second taste represented on that vertex). In the bottom row the prevalence map of two representative subjects are reported overlapped to their original anatomy.

Consequently, CBA was performed, and prevalence maps were built, only for the right hemisphere. The group prevalence map (i.e. obtained from the group-level averaging of CBA normalized individual beta maps) is shown in Fig 4 (top row).

In this representation, each vertex of the CBA normalized insular brain is assigned a color code according to taste preference and a color saturation proportional to the percentage difference between the prevalent taste and the second one in the taste ranking (e.g., a 100% value indicate that the first ranked taste is twice the second ranked taste represented on that vertex). This means that the resulting prevalence map is strictly valid only for the number and quality of tastes represented here. i.e., if we exclude one or more mapped stimuli, or if we add other possible tastes such as, e.g., fat [31], regions that are occupied by one taste would be replaced by others, possibly changing the relative positions of clusters in the resulting representation. However, from the graphic representation of the prevalence map, it is also evident how the most prevalent taste is generally almost doubling the second taste in the ranking, indicating a robust ranking for the prevalent tastes even in the presence of individual differences.

From posterior to anterior, taste prevalence clusters in the insular cortex show the following order: sweet, bitter, CO_2_, salt, umami and sour. Moreover, clusters processing sweet and bitter tastants appear coupled together in the posterior insula, whereas clusters processing umami, sour and salt appear grouped in the anterior insula, with CO_2_ processing falling in-between the sweet/bitter and umami/sour/salt groups. This pattern is also present at the single subject level, as can be appreciated in the bottom row of Fig 4, where the preference maps of two representative subjects overlaid on their own anatomy are represented. Particularly, the sweet/bitter and umami/sour coupling are preserved.

**Fig 5:**
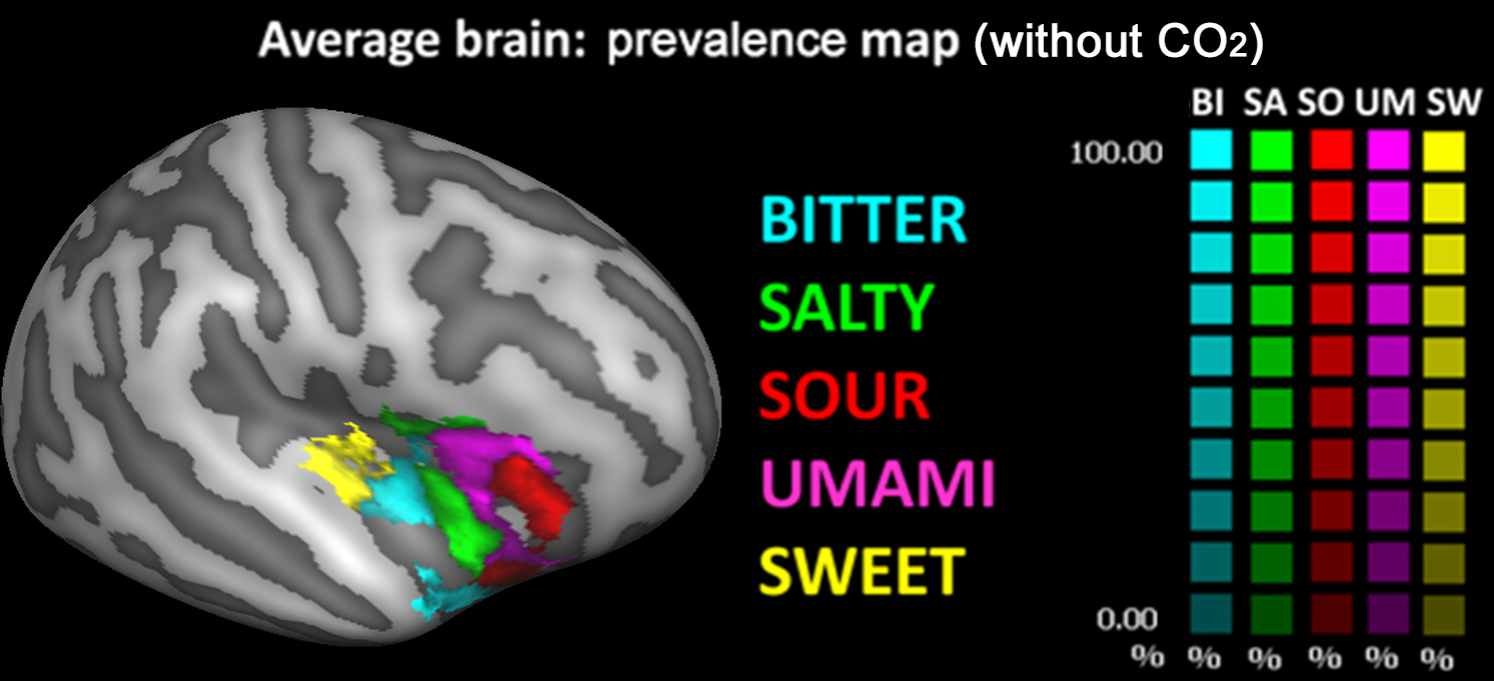
Cortical surface representation of right insular activation of the standardized five basic tastes without CO_2_. In these maps, each vertex of the CBA alignment is colored with the taste that has the biggest response averaged across all subjects and its color intensity indicate how strong is the taste prevalence (a 100% value indicate that the taste prevalence is twice the second taste represented on that vertex).

In Fig 5, the same preference map is shown after eliminating the CO_2_ data and plotting the data only for the other five tastants. This map shows that a portion of the CO_2_ cluster is assigned to the bitter cluster, whereas another portion is assigned to the salty cluster. Thus, despite the elimination of the CO_2_, a further portion of the CO_2_ cluster (located more anteriorly and inferiorly in the insular cortex) is not assigned to any of the other tastants as none of the insular responses to the other tastants exhibits a clear prevalence over the other.

**Fig 6:**
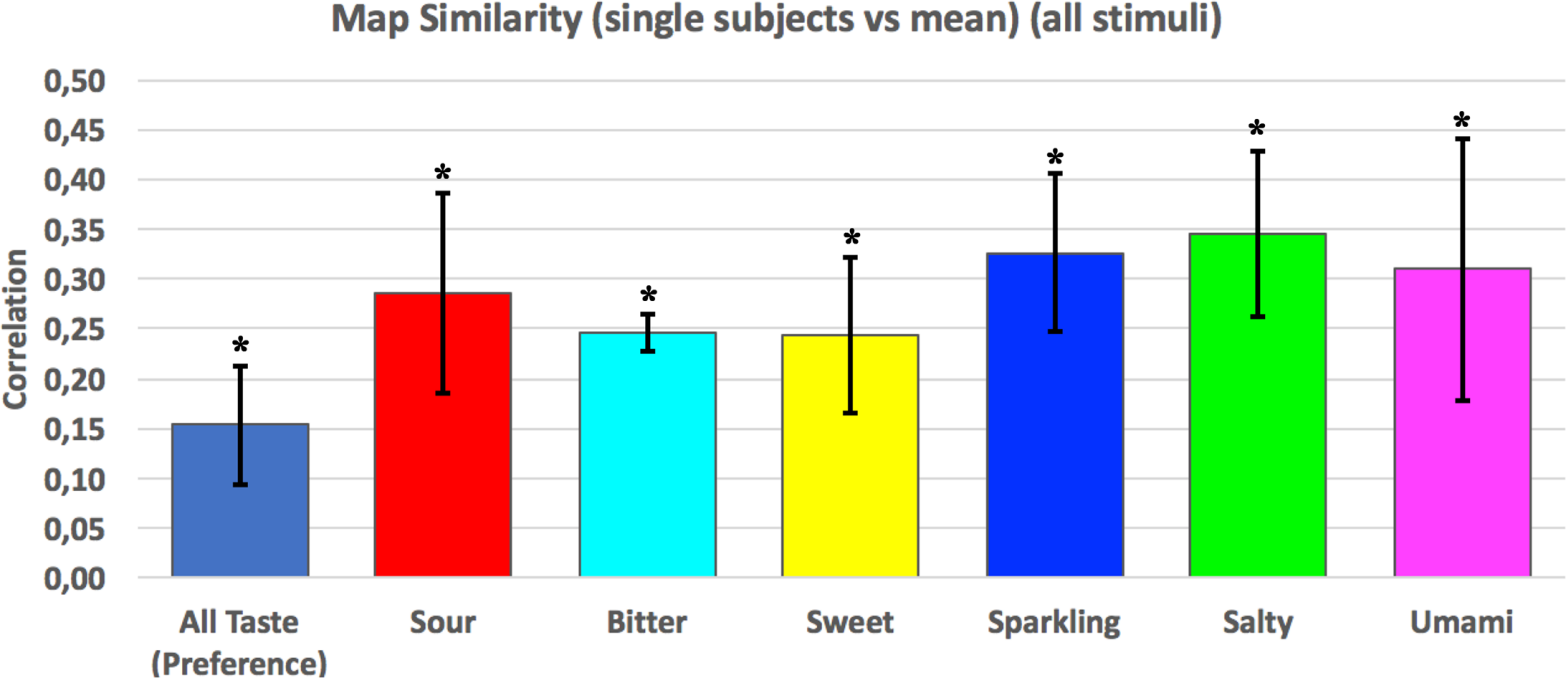
Bar graphs of the similarity of each individual subject to each and any specific tastant. In the map results of the correlation analysis of the prevalence maps of each subject vs mean prevalence maps (Spearman correlation coefficient p<0.0001) is depicted in the first bar while the other bars show the correlation of the beta maps of all the subjects for each tastant (Pearson correlation coefficient p<0.0001).

Results of the spatial correlation (similarity) analysis for each individual subject for each and any specific tastant are shown in Fig 6. As shown, the correlation was significantly positive (p<0.0001) for the responses to each individual tastant as well as for the response to all tastants (preference map with all tastes).

## Discussion

As a general rule, sensory cortices represent the complexity of the sensory world in an ordered way, with an ecologically relevant feature of the external stimulus reflected in the cortical organization along space. For instance, in the visual cortex the ecologically relevant feature is the spatial position of external stimuli that ensures a bi-univocal correspondence between the retinal and the cortical space (retinotopy). This implies that our brain can process hierarchically ordered representations of physical objects, and *operate* into the visual scene being represented, for instance programming a motor act, while body and limbs *move* into the real world. Similarly, in the auditory cortex, sounds are orderly represented on the basis of their frequency (tonotopy) and this is essential to separate sound patterns pertaining to different auditory sources. This allows, for instance, to distinguish predators from other animals, or different vehicles from each other, producing specific patterns of behavior to react to specific environmental conditions.

The subject of the present study is to analyze whether an ordered cortical representation of fundamental tastes exists in the insular cortex, which is the primary localization of taste activations [30], and whether an ecological criterion of cortical organization, similarly to other sensory cortices, also exists for the perception of taste [32].

We focused our analysis on PTC, a multisensory region involved in the neural coding and discrimination of taste qualities [33], and determined the best-stimulus responses of tastes in a group of eight subjects to generate a chemotopic map of the five basic taste modalities with (and without) CO_2_. All tastant solutions were prepared at concentrations carefully chosen to be clearly distinguishable, while remaining below the saturation threshold to avoid sense of disgusting. While it is well known that pleasantness plays a fundamental role in the cortical responses to tastes [34], here we chose to focus on taste qualities regardless of their pleasantness, as this attribute, leading to taste discrimination, is presumably of paramount importance in the brain processing of taste [33]. Taking into account the multisensory nature of PTC cells' responses and the co-occurrence of possible neural processes regarding the concentration of stimuli and their subjective appetitive or aversive valence, we measured, for each insular location, the taste eliciting the highest positive deviation from the mean insular activation, across all subjects. The resulting cortical map exhibited a clear spatial organization of all stimulated tastes, including CO_2_, yielding a chemotopic representation of taste prevalence in PTC. Taste prevalence clusters were localized in the anterior and posterior parts of the right insular cortex, without involvement of the far anterior region, in accordance with the literature [35, 36]. Although this representation is far from indicating an anatomical separation of taste qualities by neural activity (i.e., different cells respond to different tastes), the spatial organization of tastes indicates that, in our small sample, tastes tend to be preferentially localized in the same insular regions in all the subjects, whereas previous works did not report a common pattern of activation describing cortical taste preference in all examined subjects. For example, Schoenfeld et al. [14] reported a taste-specific pattern in the PTC of each of the six analyzed subjects, albeit with substantial overlap among the five basic tastes, but did not report a common taste pattern among the analyzed subjects. Therefore, we hypothesize that the differences in the anatomy of the highly folded insular cortex among subjects might explain the different results, but also that our group analysis might have benefited from the use of the cortical alignment procedure that improves the matching of each individual sulcus among subjects. Moreover, in the present work we used an analysis approach which was previously successfully used to demonstrate the tonotopic organization of the human auditory cortex at 7 Tesla [22]. Although we could not benefit of the spatial resolution and contrast-to-noise of an ultra-high magnetic field, our prevalence map showed anyway a clusterization of the five basic tastes (and CO_2_). However, it should be also pointed out that our preference map expressly displays only the “best” tastant at each vertex, thereby discarding the information about the ranks of the other tastants in each location and implicitly avoids taste overlapping in the resulting map. Future studies at ultra-high magnetic field strength are desirable to confirm this chemotopic organization of the right insular cortex, which would then represent a counterpart, for tastes, of the topological representation of acoustic tones.

While modulated by subjective preferences, tastes are associated with an innate appetitive or aversive valence and are able to evoke consequent patterns of behavior. Sweet and umami convey an appetitive valence and can drive individuals toward essential nutrients, whereas bitter and sour are associated to an aversive valence, alerting individuals against potentially harmful substances. Looking at the distribution of the five basic tastes in our prevalence map, we observed the presence of two couples of tastes with opposite valence, namely a bitter-sweet and an umami-sour couple, with salt in between. This spatial distribution may be driven by an ecological aim to facilitate the discrimination between appetitive and aversive substances. Particularly, sweet and bitter tastes evoke clearly different patterns of behavior, leading to either food acceptance (sweet) or food rejection (bitter), increasing the value of discriminating between the two classes of tastants.

On the other hand the cortical proximity of umami to salt and sour clusters, as well as the remoteness to sweet and bitter clusters is well suited to influence the perception of salt and sour stimuli. The most representative umami taste stimulus, glutamate, is a salt of glutamic acid, which has been observed to enhance the palatability of both moderately salty and sour tasting food [37] without affecting either sweet or bitter tastes.

The triplet *salt-umami-sour* would couple an appetitive taste (umami) to aversive tastes (salt and sour) allowing specifically differentiated behavioral responses. Indeed, the increase in concentration of salt above 300mM converts an innately appetitive stimulus (related to molecules necessary to maintain muscle contraction, action potentials and many other physiological functions), into a powerful aversive one [38, 39]. In an ecological perspective, the specific ability of umami to influence salt perception may be active in preventing the consumption of excessive amounts of salt, which would critically alter the electrolyte balance, while providing poor or virtually no nutrient content.

A separate consideration deserves the role played in this context by CO_2_, which is characterized by a trigeminal stimulation. To further investigate the possible non-tastant nature of CO_2_ we built the prevalence map both with and without CO_2_. Comparing these two maps (Figs 4 and 5), it was evident that a small portion of the CO_2_ cluster is not assigned to any other tastant. This might either indicate the possible “tastant” nature of CO_2_ or just represent a different activation response via the trigeminal, rather than the gustatory, pathway. Future studies using different trigeminal activators are needed to verify this hypothesis. On the other hand, the CO_2_ cluster representation close to the sweet-bitter couple also nicely fits previous findings showing a modulation of CO_2_ on the neural processing of sweet beverages with a resultant overall decrease of neural activity in sweetness-related signal processing [40]. In future works, it will be interesting to assess whether CO_2_ has on bitter beverages the same effect demonstrated on sweet beverages. Moreover, an in-depth characterization of processing levels of taste activation along the entire gustatory neuraxis, e. g. via the study of all brain networks involved in taste and CO_2_ processing, will further clarify the role of the CO_2_ beyond the organization of the primary gustatory cortex.

Apart from the small sample size, some limitations of this preliminary study need to be highlighted. First, we did not take into account the hedonic value of the tastants to verify how much of the spatial organization of the measured insular activity specifically reflects taste quality independent of this factor [34], although it is to be expected that tastants that share positive valence (sweet, umami and salt at our concentration) and negative valence (bitter and sour) in general, should agree with the obtained cortical organizational principle. Second, some of the used tastants (citric acid, CO_2_ and salt at high concentration) might induce a trigeminal response that could overlap with taste quality response, which could not be assessed in the present dataset. Third, the use of intensity-matched stimuli across the subjects would be desirable to control the effect of tastant concentration, albeit the spatial normalization of PTC responses prior to best-stimulus mapping ensured that the obtained taste preference maps were independent of the overall intensity of perception stimulated by each tastant. Moreover the use of a higher salt concentration, above the threshold that transforms appetitive valence of the stimulus into an aversive one, needs to be explored in future studies, since it might influence quality perception.

In conclusion, this study represents the first human evidence of a cortical representation of six basic tastes in the right insular cortex. The observed representation suggests the possible ecological purpose in the organization of the taste cortex, consisting in discriminating between safe nutrients and potentially harmful substances. Future works will be needed to confirm results on larger groups and to explore the patterns of response to other putative basic tastes, like fatty acid [31], pungency (also termed spiciness or hotness), coolness, calcium or metallicness. Moreover, it will be of interest to verify whether this taste spatial organization scheme of the human PTC is maintained or altered in pathologies like eating disorders or taste deficiencies.

## Supporting information

S1 File VAS questionnaire (translated in English). The file contains the Visual Analogue Scale Questionnaire that was presented to the subjects during the Behavioral Test (translated in English).
(PDF)

S2 File VAS questionnaire (original Italian version). The file contains the Visual Analogue Scale Questionnaire that was presented to the subjects during the Behavioral Test (original Italian version).
(PDF)
