# Supplementary material for: Cortical representation of different taste modalities on the gustatory cortex: A pilot study
Source: PLoS One. 2017 Dec 27;12(12):e0190164. doi: 10.1371/journal.pone.0190164 (PMC5744997; doi:10.1371/journal.pone.0190164)

TASTE PERCEPTION EVALUATION

Indicate the perceived taste with a cross and fill the VAS with a vertical line according to the perceived degree of perception

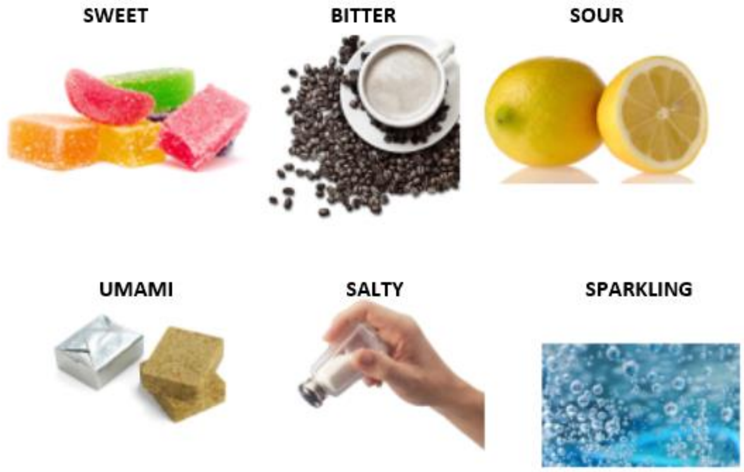

1) Which of the following tastes did you perceive after swallowing the first solution?

A. Sweet
B. Bitter
C. Sour
D. Umami (similar to stock cube)
E. Salty
F. Sparkling

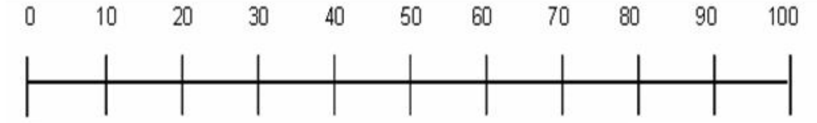

2) Which of the following tastes did you perceive after swallowing the second solution?

A. Sweet
B. Bitter
C. Sour
D. Umami (similar to stock cube)
E. Salty
F. Sparkling

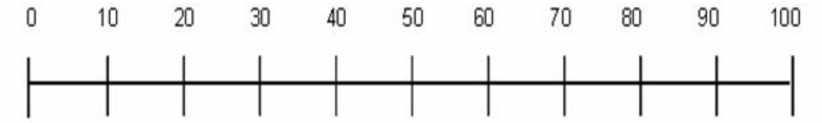

3) Which of the following tastes did you perceive after swallowing the third solution?

A. Sweet
B. Bitter
C. Sour
D. Umami (similar to stock cube)
E. Salty
F. Sparkling

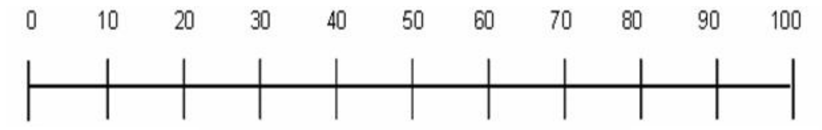

4) Which of the following tastes did you perceive after swallowing the fourth solution?

A. Sweet
B. Bitter
C. Sour
D. Umami (similar to stock cube)
E. Salty
F. Sparkling

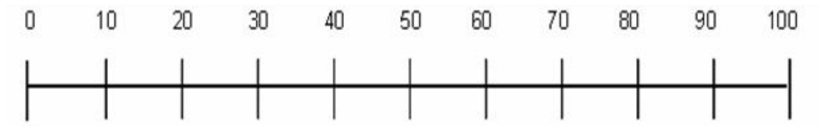

5) Which of the following tastes did you perceive after swallowing the fifth solution?

A. Sweet
B. Bitter
C. Sour
D. Umami (similar to stock cube)
E. Salty
F. Sparkling

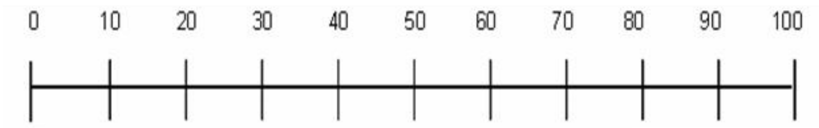

6) Which of the following tastes did you perceive after swallowing the sixthsolution?

A. Sweet
B. Bitter
C. Sour
D. Umami (similar to stock cube)
E. Salty
F. Sparkling

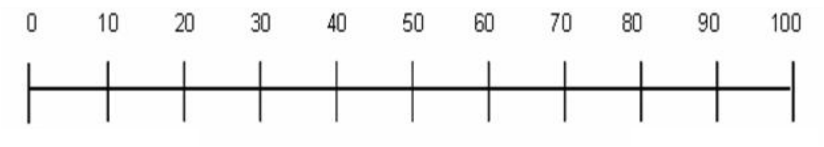

Supplement: S1 File — The file contains the Visual Analogue Scale Questionnaire that was presented to the subjects during the Behavioral Test (translated in English). (PDF) [file pone.0190164.s001.pdf]
