# Supplementary material for: Cortical representation of different taste modalities on the gustatory cortex: A pilot study
Source: PLoS One. 2017 Dec 27;12(12):e0190164. doi: 10.1371/journal.pone.0190164 (PMC5744997; doi:10.1371/journal.pone.0190164)

VALUTAZIONE PERCEZIONE GUSTATIVA

Indicare con una crocetta il gusto percepito e compilare la VAS con una linea verticale secondo il grado di percezione sentito

DOLCE

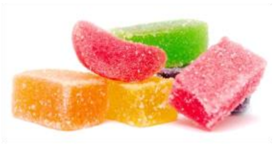

AMARO

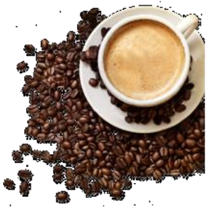

ACIDO

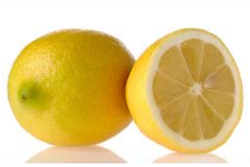

UMAMI

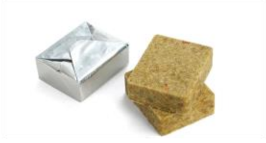

SALATO

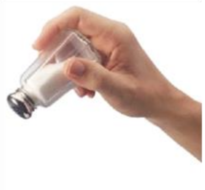

FRIZZANTE

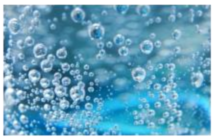

1) ***Quale dei seguenti gusti hai percepito in seguito alla deglutizione della prima soluzione?***

A. Dolce
B. Amaro
C. Acido
D. Umami (tipo dado da brodo)
E. Salato (tipo sale da cucina)
F. Frizzante

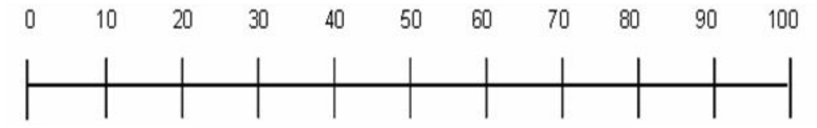

2) ***Quale dei seguenti gusti hai percepito in seguito alla deglutizione della seconda soluzione?***

A. Dolce
B. Amaro
C. Acido
D. Umami (tipo dado da brodo)
E. Salato (tipo sale da cucina)
F. Frizzante

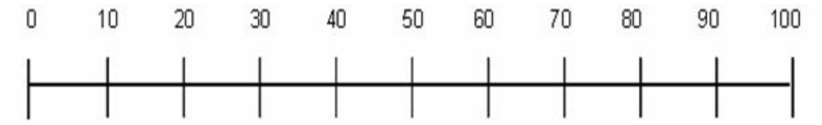

3) Quale dei seguenti gusti hai percepito in seguito alla deglutizione della terza soluzione?

A. Dolce
B. Amaro
C. Acido
D. Umami (tipo dado da brodo)
E. Salato (tipo sale da cucina)
F. Frizzante

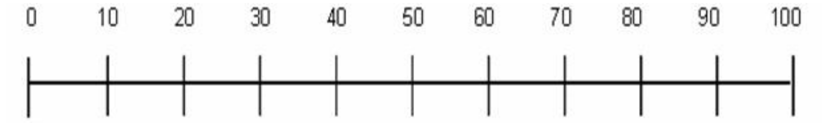

4) Quale dei seguenti gusti hai percepito in seguito alla deglutizione della quarta soluzione?

A. Dolce
B. Amaro
C. Acido
D. Umami (tipo dado da brodo)
E. Salato (tipo sale da cucina)
F. Frizzante

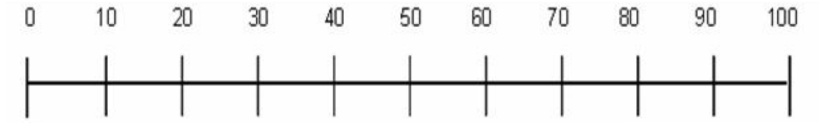

5) Quale dei seguenti gusti hai percepito in seguito alla deglutizione della quinta soluzione?

A. Dolce
B. Amaro
C. Acido
D. Umami (tipo dado da brodo)
E. Salato (tipo sale da cucina)
F. Frizzante

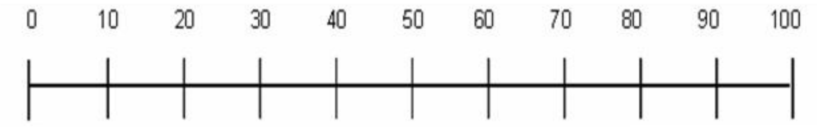

6) Quale dei seguenti gusti hai percepito in seguito alla deglutizione della sestasoluzione?

A. Dolce
B. Amaro
C. Acido
D. Umami (tipo dado da brodo)
E. Salato (tipo sale da cucina)
F. Frizzante

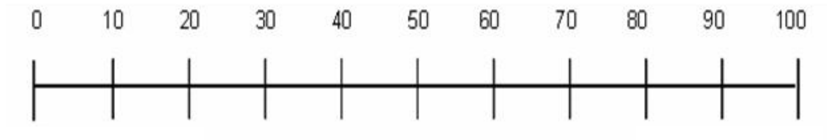

Supplement: S2 File — The file contains the Visual Analogue Scale Questionnaire that was presented to the subjects during the Behavioral Test (original Italian version). (PDF) [file pone.0190164.s002.pdf]
